# Restoration of ecosystem services in tropical forests: A global meta-analysis

**DOI:** 10.1371/journal.pone.0208523

**Published:** 2018-12-27

**Authors:** Carolina Y. Shimamoto, André A. Padial, Carolina M. da Rosa, Márcia C. M. Marques

**Affiliations:** 1 Laboratório de Ecologia Vegetal, Departamento de Botânica, Setor de Ciências Biológicas, Universidade Federal do Paraná, Curitiba, Brazil; 2 Laboratório de Análise e Síntese em Biodiversidade, Departamento de Botânica, Setor de Ciências Biológicas, Universidade Federal do Paraná, Curitiba, Brazil; University of Oregon, UNITED STATES

## Abstract

To reverse the effects of deforestation, tropical areas have expanded restoration efforts in recent years. As ecological restoration positively affects the structure and function of degraded ecosystems, understanding to what extent restoration recovers ecosystem services (ES) is an important step in directing large-scale restoration actions. We evaluated the effect of restoration in increasing the provision of ES in tropical forests. We performed a global meta-analysis of ecological indicators of the ES provided in restored areas, degraded areas and reference ecosystems. We tested for the effects of different restoration strategies, different types of degradation and for the effects of restoration over time. Overall, restoration actions contributed to a significant increase in levels of ecological indicators of ES (carbon pool, soil attributes and biodiversity protection) compared to disturbed areas. Among the restoration strategies, the natural regeneration was the most effective. Biodiversity protection and carbon recovered better than soil attributes. All other restoration strategies recovered ES to a substantially lesser degree, and reforestation with exotics decreased the ES of areas degraded by agriculture. In areas degraded by pasture, restoration was more effective in recovering the biodiversity protection, whereas in areas degraded by agriculture, the restoration recovered mainly the carbon pool. Our results show that by choosing the correct strategy, restoration can recover much of the ES lost by the degradation of tropical forests. These results should be considered for large-scale conservation and management efforts for this biome.

## Introduction

Tropical forests house approximately two thirds of the planet's terrestrial biodiversity [1] and provide many ecosystem services (ES) that are essential to human well-being [1–3], such as CO_2_ fixation [4,5], water supply and flood control [6], soil maintenance and ecotourism [2,7]. However, tropical forests are experiencing historically high levels of deforestation, with approximately 100 million hectares lost in recent years [8]. The conversion of forest areas into agricultural lands and pastures [2,9,10] and the global expansion of commercial agriculture or agribusinesses (e.g., cattle ranching, soybean farming and oil palm plantations) have led to high levels of tropical deforestation [11,12] and caused a drastic loss of ecosystem services for millions of people [13–15]. Thus, the conservation and recovery of ecosystem services in tropical forests are strategies for guaranteeing sustainability at a global level.

To reverse the effects of deforestation, tropical areas have been experiencing expanding reforestation efforts in recent years [10,16,17]. Global and large-scale initiatives such as the Bonn Challenge [18] and Initiative 20x20 [19] have set audacious targets for the restoration of degraded ecosystems (350 million hectares worldwide by 2030 and 20 million hectares in Latin America and the Caribbean by 2020, respectively), reflecting the possible increase in the area of restored ecosystems. Restoration actions focus on the recovery of the structure, function and biodiversity of degraded ecosystems [20–22]. Recently, restoration projects have systematically focused on the recovery of ecosystem services [23–25], attracting financial support [26,27]. Therefore, restoration better represents the goals for biodiversity conservation and maintenance of the ecosystem services necessary for human welfare [23,24].

Many studies have demonstrated that ecological restoration provides critical ecosystem services [25,28]. For example, ecological restoration improves soil conditions by retaining and delivering nutrients to plants, affecting nutrient and biogeochemical cycles [29–32]. Additionally, the incorporation of biomass by active or passive restoration potentially increases carbon sequestration [16,33], affecting the global carbon cycle. Restoration of degraded ecosystems is one of the most important tools for recovering biodiversity [34]. Although one-off studies point to the importance of restoration to recover ES in tropical forests, the impacts of these effects on a broad scale are scarcely known.

Restoration can be performed with different strategies (natural regeneration, nucleation, reforestation with native, reforestation with exotic, agroforestry), and the choice of strategy is based on a set of local features, including ecological (ecosystem resilience, land use, landscape descriptors), social (possibility of providing income for local communities) and economic (management costs) features [20,35–38]. Little is known about how the choice of the restoration strategy potentially affects the effectiveness in recovering ecosystem services [39]. Ecological restoration can also be affected by the type of disturbance in the target area: its success will depend on the intensity, extent, severity and duration of land use [37,40,41]. In this context, different types of disturbances, such as human impacts (logging, agriculture, pasture) and natural disturbance (tree-falls, hurricanes, landslides), affect many site-specific factors that influence the recovery rate of ecosystem services [36,42]. Moreover, the rates of recovery of ecosystem services can be influenced by the time since abandonment [25] and the landscape context [43]. In each successional stage, biotic factors (species composition) and abiotic factors (soil nutrients) could change and generate particular services [37]. Many studies analyze the change in these factors in chronosequences [44–47], but evaluating the influence of time on the recovery of each type of ecosystem service from its respective restoration strategy is also necessary. Thus, to what extent the restoration strategy choice and the type of degradation affect the restoration of ecosystem services is crucial for the conservation and management of tropical forests. Some previous meta-analysis studies in tropical forests approached specifically the drivers of the success of restoration [43], the effects of restoration strategies [48], and the changes in specific ES [34,49]. However, it is not still clear how the restoration in areas with different degradation history affect multiple ES along the time.

In this study, we evaluated the effects of restoration in increasing the provision of ES in tropical forests. We performed a comprehensive search for restoration studies and conducted a meta-analysis of ecological indicators of ecosystem services provided in restored and degraded areas and reference ecosystems of tropical forests across the globe. Specifically, we tested whether (1) the restoration strategy and the type of degradation affect the amount of ES in the restored area compared to degraded and reference areas; (2) ecosystem services recover differently; and (3) ES increase over time after the restoration has been initiated.

## Methods

### Obtaining data

We searched the scientific literature to identify quantitative measures of variables (ecological indicators) related to the provision of one or more ecosystem services in tropical forests. As biodiversity protection has been considered an ES [50], we also used biodiversity metrics (see below) as ecological indicators. The selection was restricted to studies that comparatively presented quantitative measures of levels of ecological indicators of ES in one of the following conditions: (i) degraded and restored; (ii) restored and reference ecosystem; or (iii) degraded, restored and reference ecosystems. We defined the degraded area as the starting point of restoration, the restored area as being directly or indirectly subjected to restoration actions and the reference ecosystem as the undisturbed area. The search criteria included studies conceived as ecosystem restoration projects (for example, reforestation with native or exotic species, nucleation, natural regeneration), studies designed to maximize forest production (agroforestry) or studies that comparatively surveyed abandoned areas where natural regeneration occurred. In all situations, ecological indicators were locally measured and used for comparisons.

**Fig 1:**
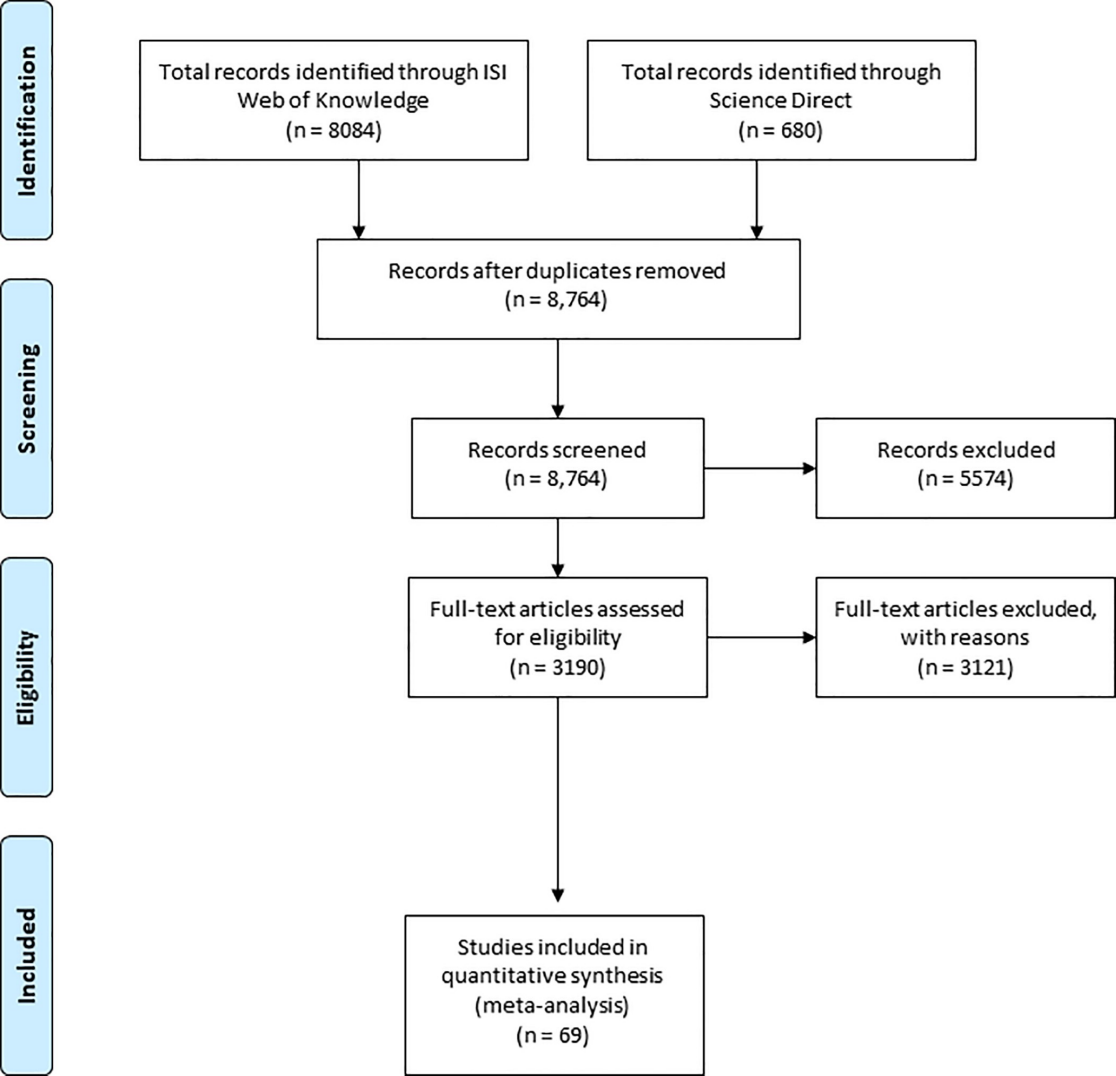
Flow diagram of database searching and article screening. The checklist of the total Prisma 2009 requirements is in S2 Table.

We conducted the search in the scientific databases ISI Web of Knowledge and Science Direct using the following terms and combinations, without restriction to year (until May 2017): (tropical* forest) AND (restoration* OR regeneration* OR recuperation* OR rehabilitation* OR restore* OR recovery* OR reforestation native* OR sucession* OR disturbance* OR perturbation). The preliminary search was limited to the following subject areas “climate change”, species richness”, “costa rica,national park”, “tree species”, “tropical forest”, “ecosystem service”, “soil,microbial biomass”, “organic matter”, “Brazil”, “forest ecology”, “atlantic forest”, “microbial community”, “soil organic carbon”, “forest management”, “secondary forest”, “puerto rico" in Science Direct search. With these terms, we obtained 8,764 articles including studies in tropical and subtropical forests. In a prior analysis of the title and summary of each study, we selected 3,190 articles that contained all quantitative variables necessary for the meta-analysis. Then, we searched each article for quantitative variables (mean, standard deviation, sample size and age) of ecological indicators in degraded, restored and/or reference conditions (see below). From this search, 69 articles from 25 countries in five continents were found (Fig 1; Supporting information S1 Table; S1 Fig).

For each study, we compiled ecological indicators based on Benayas *et al*. [51] and respective ecosystem services, according to MEA [2], as follows: carbon pool (aboveground biomass, below-ground biomass, dead organic matter and soil organic carbon) and soil attributes (C, Ca, Mg, N, Nitrate, P, pH, cation exchange capacity [CEC], water holding capacity, and soil organic matter). Moreover, we compiled species richness, diversity, density and abundance data as a proxy for evaluating the effect of restoration practices on recovering biodiversity protection (Table 1).

**Table 1:** Definition and number of ecological indicators (n) of ecosystem services of parameters considered in the meta-analysis. *CEC: cation exchange capacity.

Parameter	Definition	n
**Restoration strategy**		
Natural regeneration	Passive or assisted restoration	307
Nucleation	Active restoration using individual trees to improve facilitation	33
Reforestation with native	Active restoration by planting native species	208
Reforestation with exotic	Active restoration by planting exotic species	158
Agroforestry	Active restoration by planting economically important species (*Pinus spp*, *Eucalyptus spp*, *Acacia spp and Tectona spp*) or native species and agricultural crops	165
*Total*		866
**Degradation type**		
Pasture	Pasture from 10 to 25 years	109
Logging	Tree removal	6
Agriculture	Plantation of food crops such as coffee, mustard and vegetables for 5 to 40 years	273
*Total*		389
**Ecosystem service**		
Carbon pool	Aboveground biomass,belowground biomass, soil, dead and soil organic carbon	73
Soil attributes	Nutrients (C, Ca, Mg, N, Nitrate, P), pH, CEC*, soil organic matter and water holding capacity	465
Biodiversity protection	Species richness, density and abundance of plants, birds, lizards, frogs, termites, ants and worms	333
*Total*		866

From these 69 studies, we extracted 866 quantitative measures of ES: 383 independent effect sizes were calculated comparing restoration and degraded areas, and 685 independent effect sizes were calculated comparing restoration areas and reference ecosystems. To explain heterogeneity in effect sizes, we compared the effect sizes among five restoration strategies (natural regeneration, nucleation, reforestation with native species, reforestation with exotic species and agroforestry) and three types of land use (i.e., degradation) prior to restoration (pasture, logging and agriculture) (Table 1). When the sample size was sufficient (pasture and agriculture), we compared the effects of different restoration strategies on ES recovery in the degraded ecosystem. For the land use “logging” the sample size was insufficient for analysis.

### Data analysis

We extracted the mean, standard deviation and sample size for each ecological indicator of ES in the primary studies. Using this information, we calculated Hedges’ *g* effect size, the variance and the bootstrap confidence interval (CI). Hedges’ *g* (average differences divided by standard deviation) is a variation of Cohen’s *d* that includes a correction of deviations, which are derived from a small sample [52]. According to our criteria for estimating effect sizes, a positive value means that the amount of ES in restored areas is higher than in degraded areas or a reference ecosystem; a negative value means the opposite.

Before summarizing the effect sizes to obtain an overall effect of restoration, we tested the hypothesis of real heterogeneity among studies using *Q-*statistics [53]. Our data were considered heterogeneous (*P* (*Q*) ≤ 0.05), and therefore, random effects models were used to calculate the average effect size (*g*+). We also tested if heterogeneity among studies could be explained by the type of restoration and the type of degradation using subgroup analyses [52]. Similarly, we performed meta-regression analyses [52] between effect sizes and restoration time to analyze whether the efficacy of ES recovery depended on the time since restoration began.

Additionally, we performed a complementary analysis in order to detect any bias in the effect sizes in the metanalysis. For this, we did the fail-safe N of Rosenthal [54] and of Orwin [55] analysis and, also, the trim and fill technique [56].

## Results

Overall, ecological restoration promotes an increase in ES in relation to degraded areas (*g+* = 0.37; CI: 0.16; 0.57), but less so when compared to the reference ecosystem (*g+* = -1.09; CI: -1.27; -0.96). There was no evidence of bias for both mean effect sizes above (see S2 Fig and S3 Fig). The mean effects are not an artifact of the bias, given that the number of studies required to change the effect size is the same of the actual number of effects (Orwin’s fail safe for ES in relation to degraded area: 360; and for ES in relation to the reference ecosystem: 643). Relatedly, the number of effects to change the interpretation is astronomic for both comparisons (14,004 and 295,208; respectively). Finally, the trim and fill technique did change the mean effect size only for reference-restored comparison, but never changed the interpretation of significance: *g+* = 0.37 (CI: 0.20; 0.54) and *g+* = -0.50 (CI: -0.65; -0.34) after the trim and fill, respectively.

### Effects of restoration strategies

**Fig 2:**
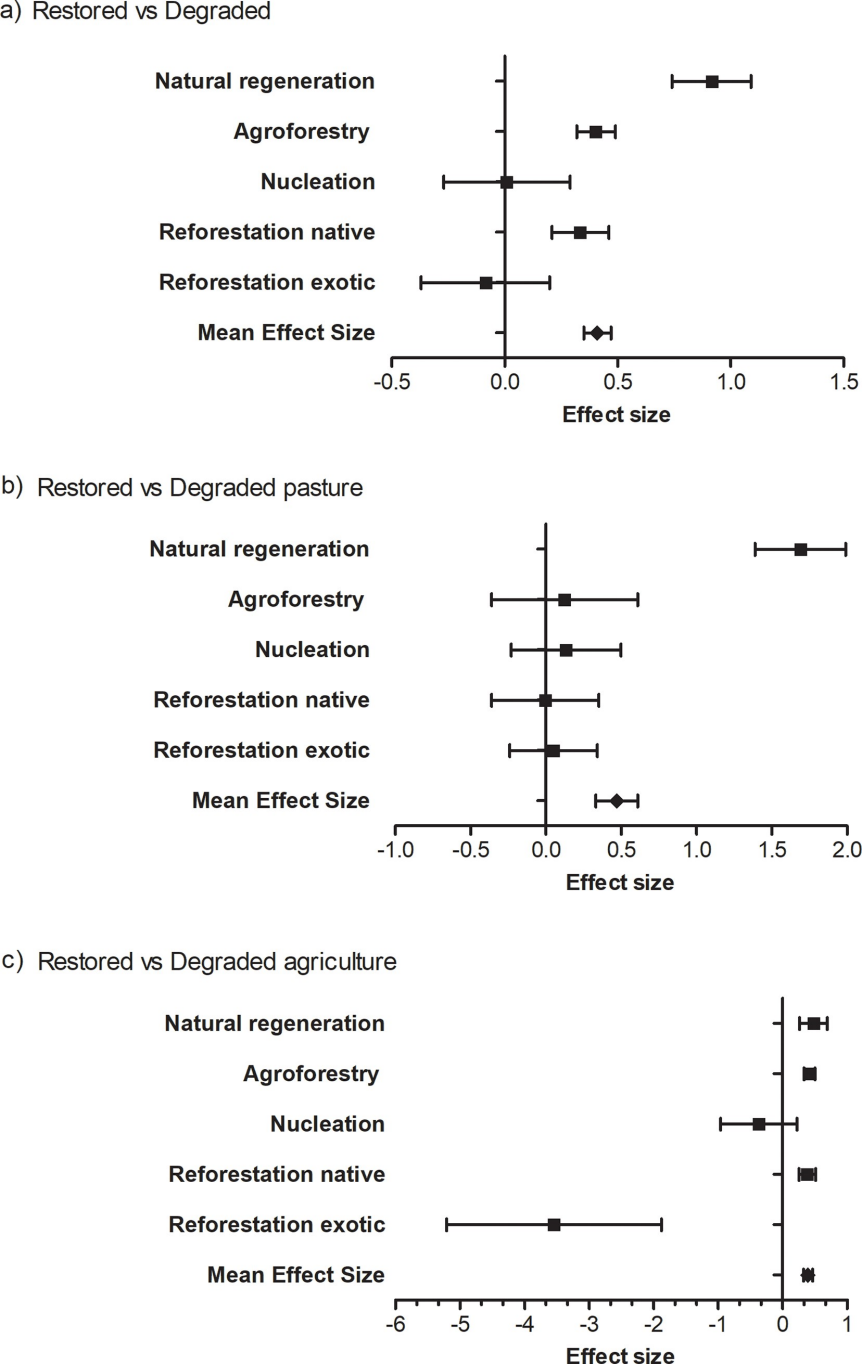
Effect size (average and bootstrap CI) of ecosystem services recovered in restoration areas, according to restoration strategies. (A) All ecosystem degradation types; (B) Degradation by pasture; (C) Degradation by agriculture. The vertical line represents the null hypothesis. Positive effect size means that the amount of ES in restored areas is higher than in degraded area.

The restoration increased the ES by in relation to degraded areas (*g+* = 0.41; *Q* = 2261.31; DF = 348; *P<*0.0001; Fig 2A). Differences in the ES recovery existed among restoration strategies (*Q* = 56.97; DF = 4; *P*<0.001; Fig 2A), with the “natural regeneration” strategy increasing mostly the ES compared to degraded areas (*g+* more than the double of the other strategies), followed by “agroforestry”, and “reforestation with native species”. ES following the “reforestation with exotics” strategy and nucleation did not differ from the degraded areas (Fig 2A).

Considering the degradation types separately (Fig 2B), the restoration also significantly recovered the ES in areas degraded by pasture (*g+* = 0.47; *Q* = 559.84; DF = 80; *P*<0.001; Fig 2B). In this situation, the restoration strategy also affected the recovery of ES (*Q* = 95.87; DF = 4; *P*< 0.001; Fig 2B), and “natural regeneration” was the only strategy that positively affected the ES, strongly increasing the ES (see *g+* values in Fig 2B) compared to pasture.

In the same way, the restoration recovered the ES in areas degraded by agriculture (*Q* = 1622.91; DF = 263; *P*<0.0001; Fig 2C). In such areas, the type of restoration strategy affected the ES recovery (*Q* = 38.71; DF = 4; *P*< 0.001; Fig 2B), with the “natural regeneration” strategy also mostly increasing the ES, followed by “agroforestry” and “restoration with native species”. Nucleation did not significantly affect the ES, and surprisingly, the restoration using exotics negatively affected the ES in areas degraded by agriculture (Fig 2C).

### Effects of restoration in different ecosystem service categories

**Fig 3:**
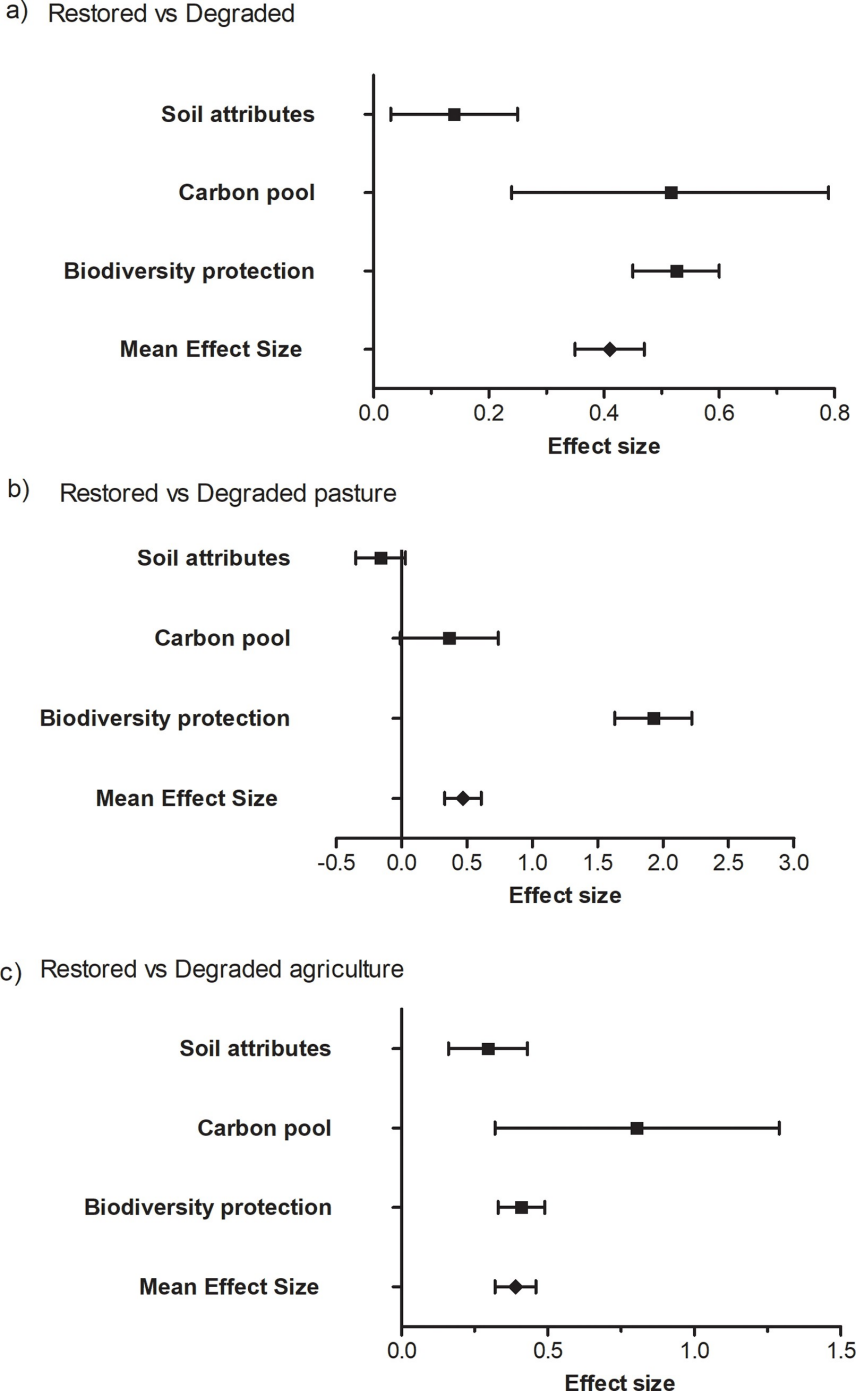
Effect size (average and bootstrap CI) of ecosystem services recovered in restoration areas, according to different types of services (soil attributes, carbon pool and biodiversity protection). (A) All ecosystem degradation types; (B) Degradation by pasture; (C) Degradation by agriculture. The vertical line represents the null hypothesis. Positive effect size means that the amount of ES in restored areas is higher than in degraded area.

Generally, the restoration positively affected all the ES types (*Q* = 2284.46; DF = 350; P < 0.001; Fig 3A). Additionally, differences existed among ES categories (*Q* = 33.82; DF = 2; *P*<0.001), with “biodiversity protection” recovering by mostly, followed by carbon pools and soil attributes compared to the degraded areas. Considering the degradation types separately, the restoration significantly recovered the ES in areas degraded by pasture (*Q* = 502.80; DF = 82; *P*<0.001; Fig 3B). There were differences among ES categories (*Q* = 152.92; DF = 2; *P*<0.001), with the “biodiversity protection” category increasing mostly compared to the areas degraded by pasture (Fig 3B). In areas degraded by agriculture, the restoration positively affected the ES (*Q* = 1656.22; DF = 265; *P*<0.001, Fig 3C), with no difference among the ES categories (*Q* = 5.40; DF = 2; P = 0.07; Fig 3C).

### The recovery of the ecosystem services along the time of restoration

**Fig 4:**
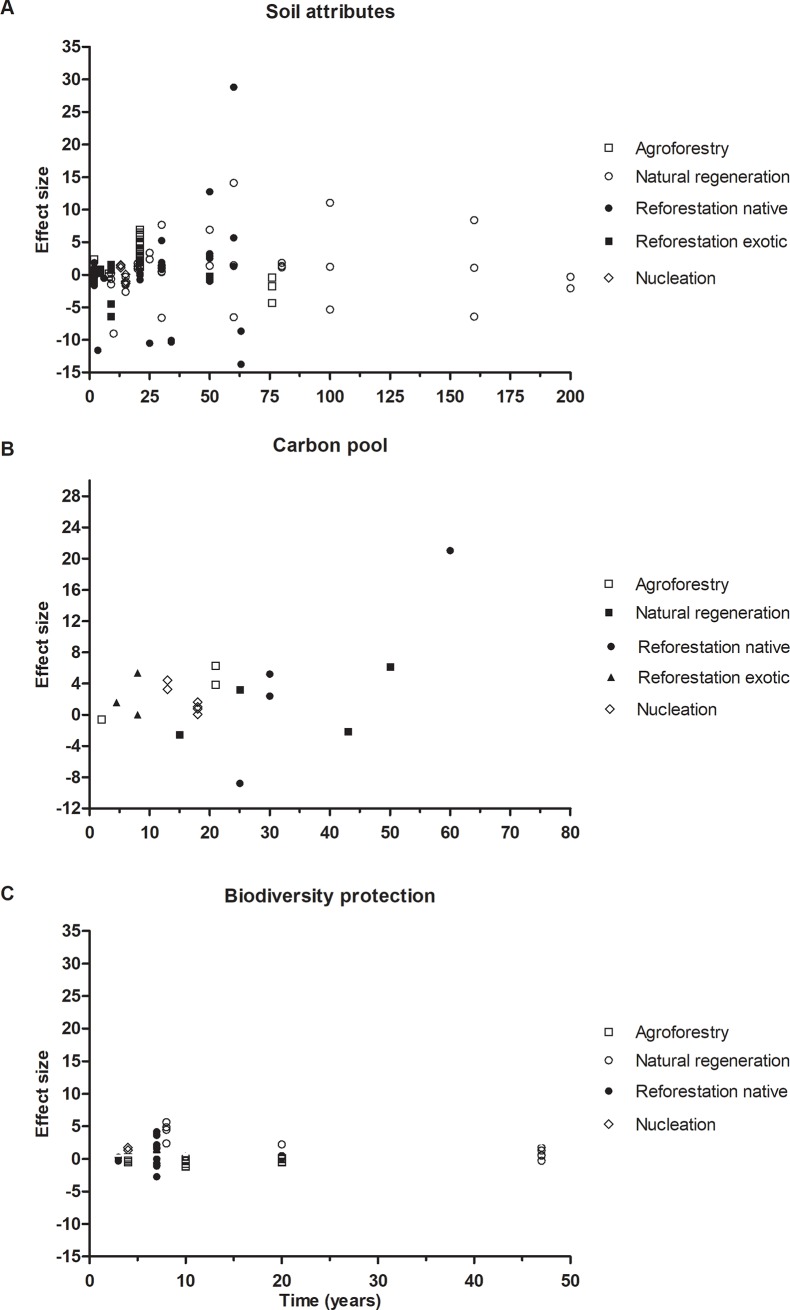
Meta-regression of age of restored ecosystem and the effect size of different ecosystem services. (A) Soil attributes (n = 150); (B) Carbon pool (n = 13); (C) Biodiversity protection (n = 71). Positive effect size means that the amount of ES in restored areas is higher than in degraded area. Three outlier was removed from (B).

The recoveries of soil attributes (*Q* = 0.86; DF = 1; *P* = 0.35; Fig 4A), carbon sequestration (*Q* = 0.0005; DF = 1; *P* = 0.98; Fig 4B), and biodiversity protection (*Q* = 0.59; DF = 1; *P* = 0.44; Fig 4C) were not significantly related to forest age.

## Discussion

We found that ecological restoration positively affects the recovery of ecosystem services (carbon pools, soil attributes and biodiversity protection) in tropical forests, increasing the ES compared to disturbed areas. This result highlights the importance of restoration as a tool to reverse the degradation of this highly threatened biome by increasing biodiversity resilience and providing important ecosystem services [57]. The reference ecosystem still has a very large proportion of ES when compared to restored areas, suggesting that preserving the remaining areas of tropical forests is more conservative in maintaining the forest functionality and services [58, 59]. Thus, given the large fragmentation and habitat losses of tropical areas [60], large-scale tropical forest conservation and management strategies should promote the conservation of remnants while encouraging active or passive restoration actions.

### Effects of restoration strategies on ES

The passive restoration by natural regeneration was the best strategy for recovering ES, increasing the ES of the degraded ecosystem by 91%. Restoration by natural regeneration can increase the carbon present in the degraded area 10-fold [33], causing a global accumulation of 8.48 PgC over 40 years [16]. Additionally, restoration by natural regeneration recovers the biodiversity higher than reforestation [34]. Natural regeneration is especially important to recovering ES in areas degraded by pasture, because in this situation, the ES are approximately three-fold greater than the ES recovered in areas degraded by agriculture. By providing such growth in ecosystem services and by being the least expensive method to recover disturbed areas [57, 60], natural regeneration is potentially important to large-scale landscape restoration in tropical regions [48,61,62]. However, this strategy is efficient only in situations where degradation is low and where the soil seed bank and the source of propagules from adjacent fragments are still present [40,41,63,64]. As there is a large variation is soil degradation and landscape characteristics along tropical region, the interpretation of this result shoud be taken carefully [65].

Agroforestry (increasing ES by 41%) and plantation of native species [34%] positively affected the ES of degraded areas. The integration of native trees and agricultural crops or livestock in agroforestry contributes to carbon sequestration [66,67], erosion control, water quality [68–72] and soil fertility [73–75]. Restoration with native species potentially recovers a large part of the ES, but specific situations such as the low diversity of species in the planting system [76] or the choice of species with a very low impact in delivering ES [77], can drastically reduce the effectiveness of the restoration. Additionally, our results showed that the ES recovered by these two strategies are strongly limited in areas degraded by pasture, restricting the effectiveness of these strategies locally.

The restoration with exotic species and nucleation strategies may not be recommended to restore tropical forests because they did not affect (or even diminished) the ES compared to degraded areas. Exotic species can promote low levels of carbon stocks, water regulation, and nutrient cycling and are generally unable to support the biodiversity of native flora [78,79]. This occurs because exotic species have faster growth rates and lower accumulation of aboveground biomass [80], and most detritus under those species is in the form of litter that translates into lower soil C storage through decomposition [81,82]. In addition, the roots of exotic species cause a disruption of the soil that reduces soil organic matter and contributes to the loss of water retention [83]. On the other hand, nucleation is generally limited in increasing the seedling recruitment of tropical forests [84], potentially having a cascading effect on all ecosystem functions.

### Restoration of different ES categories

From all analyzed ES categories, the restoration can recover much more biodiversity protection (53%) and carbon storage (52%) than soil attributes (14%) in the degraded area. Similar patterns were also found in a global meta-analysis of restored agroecosystems, which increased biodiversity by 68%, the carbon sequestration by 62%, and the supporting services by 42% (soil physical quality and soil chemical quality) [85]. The recovery of biodiversity is affected primarily by the decision of the restoration practitioner in using the largest number of species possible in the restoration project [86] or by the rapid colonization of species in areas of natural regeneration. After the first several years, the biodiversity rapidly increases under restoration from colonization of the area by other plant species, pollinators, dispersers and other animals [86,87,88,89]. Restoration actions can increase the biodiversity from 22% to 196% compared with a degraded area [88]. The focus of restoration on biodiversity is important for protecting endangered or endemic biota [78], as has been an important goal of the Aichi Targets.

The relatively high recovery of the carbon pool in the restored ecosystems can be explained by the recurrent use of fast growing species in restoration projects, which rapidly affects the aboveground biomass in tropical forests [33,49, 89–91]. Consequently, as the aboveground biomass increases, the below-ground biomass, litter deposition and organic soil carbon also increase. Carbon sequestration is important for minimizing the effects of global warming [92,93] and contributes to achieving the goals of lowering carbon levels in the atmosphere established in large-scale agreements such as the Bonn Challenge and Initiative 20x20.

The contrasting recovery pattern of carbon pools and biodiversity protection among areas degraded by pasturing and agriculture is possibly a differential effect of these degradation types on the ecosystem properties. Degradation by pasture affects the soil porosity and permeability, restricting the plant growth, productivity and biomass in restoration areas [94], while biodiversity is probably less affected. On the other hand, areas degraded by agriculture potentially limit the biodiversity because of the pervasive effect of pesticides on the biota [95], while the biomass and carbon pool are rapidly recovered in the restoration areas. Similar results were found in a review of tropical reforestation that showed that forests growing on abandoned agricultural land accumulate more carbon and quickly compared to pastures [96].

### Effects of restoration time

The predicted gradual increase of different ES along with restoration time was not confirmed in our study. Studies have theorized that the renewal of biodiversity and ecosystem services during restoration follows a asymptotic curve, with a marked increase in the early years and a tendency to stabilize over time [97]. On a broader temporal (up to 200 years) and spatial (tropical global) scale, and considering multiple ES, this progressive increase was not revealed, suggesting a continuous (but not progressive) incorporation of ES into the restoration ecosystem. Although we have evaluated a limited number of ecosystem services, the temporal independence observed here suggests that the restoration may have longer-lasting effects than previously revealed in the studies. This should be considered in large-scale restoration policy agendas.

In general, we found that different ecological restoration actions promote an increase in ES in relation to degraded areas, but the ES are still far from those of the reference systems. Restoration actions are a good response to forest degradation because they recover the structure and function of these areas. However, some services can take 50–80 years to be recovered, while other can never reach the values of the intact forest [49]. Thus, the conservation of remaining tropical forests should be emphasized [20,98]. Our results also demonstrate the effect of different restoration strategies in the recovery of ES. Thus, the acknowledgment of how much the restoration actions promote the recovery of ES is important for guiding the management and intervention practices in these forests. Understanding these processes can guide which type of restoration action generates the desired ecosystem services.

## Supporting information

S1 Table List of studies analyzed. (DOCX)

S2 Table Prisma 2009 checklist. (DOC)

S1 Fig Location of 69 studies from 25 countries distributed in five continents. (DOCX)

S2 Fig Funnel plot for comparison between restored and reference ecosystem. (DOCX)

S3 Fig Funnel plot for comparison between restored and degraded ecosystem. (DOCX)
