# Supplementary material for: Restoration of ecosystem services in tropical forests: A global meta-analysis
Source: PLoS One. 2018 Dec 27;13(12):e0208523. doi: 10.1371/journal.pone.0208523 (PMC6307725; doi:10.1371/journal.pone.0208523)
Supplement: S1 Table — (DOCX) [file pone.0208523.s001.docx]

S1 Table. List of studies analyzed

Aerts R, Lerouge F, November E, Lens L, Hermy M, Muys B (2008) Land rehabilitation and the conservation of birds in a degraded Afromontane landscape in northern Ethiopia. Biodiversity and Conservation 17:53–69.

Armbrecht I, Perfecto I (2003) Litter-twig dwelling ant species richness and predation potential within a forest fragment and neighboring coffee plantations of contrasting habitat quality in Mexico. Agriculture, Ecosystems and Environment 97: 107–115.

Armbrecht I, Perfecto I, Silverman E (2006) Limitation of nesting resources for ants in Colombian forests and coffee plantations. Ecological Entomology 31: 403–410.

Aryal DR, De Jong BHJ, Ochoa-Gaona S, Esparza-Olguin L, Jorge Mendoza-Veja J (2014) Carbon stocks and changes in tropical secondary forests of southern Mexico. Agriculture, Ecosystems and Environment 195: 220–230.

Ashagrie Y, Zech W, Guggenberger G (2005) Transformation of a Podocarpus falcatus dominated natural forest into a monoculture Eucalyptus globulus plantation at Munesa, Ethiopia: soil organic C, N and S dynamics in primary particle and aggregate-size fractions. Agriculture, Ecosystems and Environment 106: 89–98.

Attignon SE, Lachat T, Sinsin B, Nagel P, Peveling R (2005) Termite assemblages in a West-African semi-deciduous forest and teak plantations. Agriculture, Ecosystems and Environment 110: 318–326

Baatuuwie NB, Asare NA, Osei EMJnr, Quaye-Ballard JA (2011) The restoration of degraded forests in Ghana: a case study in the Offinso forest district. Agriculture and Biology Journal of North America 2(1): 134-142

Barlow J, Gardner TA, Ferreira LV, Peres CA (2007) Litter fall and decomposition in primary, secondary and plantation forests in the Brazilian Amazon. Forest Ecology and Management 247: 91–97

Behera N, Sahani U (2003) Soil microbial biomass and activity in response to Eucalyptusplantation and natural regeneration on tropical soil. Forest Ecology and Management 174: 1–11.

Benavides AM, Wolf JHD, Duivenvoorden JF (2006) Recovery and succession of epiphytes in upper Amazonian fallows. Journal of Tropical Ecology 22:705–717.

Beukema H, van Noordwijk M (2004) Terrestrial pteridophytes as indicators of a forest-like environment in rubber production systems in the lowlands of Jambi, Sumatra. Agriculture, Ecosystems and Environment 104: 63–73.

Bischoff W, Newbery DM, Lingenfelder M, Schnaeckel R, Petol GH, Madani L, Ridsdale CE (2005) Secondary succession and dipterocarp recruitment in Bornean rain forest after logging. Forest Ecology and Management 218: 174–192.

Boley JD, Drew AP, Andrus RE (2009) Effects of active pasture, teak (Tectona grandis) and mixed native plantations on soil chemistry in Costa Rica. Forest Ecology and Management 257: 2254–2261.

Calle Z, Henao-Gallego N, Giraldo C, Armbrecht I (2013) A Comparison of Vegetation and Ground-Dwelling Ants in Abandoned and Restored Gullies and Landslide Surfaces in the Western Colombian Andes. Restoration Ecology 21(6): 729–735.

Castro-Luna AA, Sosa VJ, Castillo-Campos G (2007) Bat diversity and abundance associated with the degree of secondary succession in a tropical forest mosaic in south-eastern Mexico. Animal Conservation 10: 219–228.

Cavelier J, Tobler A (1998) The effect of abandoned plantations of Pinus patula and Cupressus lusitanica on soils and regeneration of a tropical montane rain forest in Colombia. Biodiversity and Conservation 7: 335-347.

Chidumayo EN, Kwibisa L (2003) Effects of deforestation on grass biomass and soil nutrient status in miombo woodland, Zambia. Agriculture, Ecosystems and Environment 96: 97–105.

Chua SC, Ramage BS, Ngo KM, Potts MD, Lum SKY (2013) Slow recovery of a secondary tropical forest in Southeast Asia. Forest Ecology and Management 308: 153–160.

Deng L, Wangb K, Tanga Z, Shangguana Z (2016) Soil organic carbon dynamics following natural vegetation restoration: Evidence from stable carbon isotopes (d13C). Agriculture, Ecosystems and Environment 221: 235–244.

**Do TV, Osawa A, Thang NT (2010) Recovery process of a mountain forest after shifting cultivation in Northwestern Vietnam. Forest Ecology and Management 259: 1650–1659.**

Durães R, Carrasco L, Smith TB, Karubian J (2013) Effects of forest disturbance and habitat loss on avian communities in a Neotropical biodiversity hotspot. Biological Conservation 166: 203–211.

Fink RD, Lindell CA, Morrison EB, Zahawi RA, Holl KD (2009) Patch Size and Tree Species Inﬂuence the Number and Duration of Bird Visits in Forest Restoration Plots in Southern Costa Rica. Restoration Ecology Vol 17: 479–486.

Folt B, Reider KE (2013) Leaf-litter herpetofaunal richness, abundance, and community assembly in mono-dominant plantations and primary forest of northeastern Costa Rica. Biodiversity and Conservation 22: 2057–2070.

Fragoso C, Leyequién E, García-Robles M, Montero-Muñoz J, Rojas P. (2016) Dominance of native earthworms in secondary tropical forests derived from slash-and-burn Mayan agricultural practices (Yucatán, Mexico). Applied Soil Ecology 104: 116–124

Gahagan A, Giardina CP, King JS, Binkley D, Pregitzer KS, Burton AJ (2015) Carbon fluxes, storage and harvest removals through 60 years of stand development in red pine plantations and mixed hardwood stands in Northern Michigan, USA. Forest Ecology and Management 337: 88–97.

González-Iturbe JA, Olmsted I, Tun-Dzul F (2002) Tropical dry forest recovery after long term Henequen (sisal, Agave fourcroydes Lem.) plantation in northern Yucatan, Mexico. Forest Ecology and Management 167: 67–82

Guidi C, Vesterdal L, Gianelle D, Rodeghiero M (2014) Changes in soil organic carbon and nitrogen following forest expansion on grassland in the Southern Alps. Forest Ecology and Management 328: 103–116.

Huang Y, Li Y, Xiao Y, Wenigmann KO, Zhou G, Zhang D, Wenigmann M, Tang X, Liu J (2011) Controls of litter quality on the carbon sink in soils through partitioning the products of decomposing litter in a forest succession series in South China. Forest Ecology and Management 261: 1170–1177.

Jiménez JJ, Lal R, Leblanc HA, Russo RO (2007) Soil organic carbon pool under native tree plantations in the Caribbean lowlands of Costa Rica. Forest Ecology and Management 241: 134–144.

Kammesheidt L (1998) The role of tree sprouts in the restoration of stand structure and species diversity in tropical moist forest after slash-and-burn agriculture in Eastern Paraguay. Plant Ecology 139: 155–165.

Lagerlöf J, Adolfsson L, Börjesson G, Ehlers K, Vinyoles GP, Sundh I (2014) Land-use intensiﬁcation and agroforestry in the Kenyan highland: Impacts on soil microbial community composition and functional capacity. Applied Soil Ecology 82: 93–99.

Lee EWS, Hau BCH, Corlett RT (2005) Natural regeneration in exotic tree plantations in Hong Kong, China. Forest Ecology and Management 212: 358–366

Lee TM, Soh MCK, Sodhi N, Koh LP, Lim SLH (2005) Eﬀects of habitat disturbance on mixed species bird ﬂocks in a tropical sub-montane rainforest. Biological Conservation 122: 193–204.

Lemma B, Kleja DB, Nilsson I, Olsson M (2006) Soil carbon sequestration under different exotic tree species in the southwestern highlands of Ethiopia. Geoderma 136: 886–898.

Lemma B, Olsson M (2006) Soil δ15N and nutrients under exotic tree plantations in the southwestern Ethiopian highlands. Forest Ecology and Management 237: 127–134.

Lees AC, Moura NG, Almeida AS, Vieira ICG (2015) Poor Prospects for Avian Biodiversity in Amazonian Oil Palm. PLoS ONE 10(5): 1-17.

Leuschner C, Harteveld M, Hertel D (2009) Consequences of increasing forest use intensity for biomass, morphology and growth of ﬁne roots in a tropical moist forest on Sulawesi, Indonesia. Agriculture, Ecosystems and Environment 129: 474–481.

Lima TA, Vieira G (2013) High plant species richness in monospeciﬁc tree plantations in the Central Amazon. Forest Ecology and Management 295: 77–86.

Liu N, Ren H, Yuan S, Guo Q, Yang L (2013) Testing the Stress-Gradient Hypothesis During the Restoration of Tropical Degraded Land Using the Shrub Rhodomyrtus tomentosa as a Nurse Plant. Restoration Ecology 21: 578–584.

Lugo AE (1992) Comparison of Tropical Tree Plantations with Secondary Forests of Similar Age. Ecological Monographs 62: 1-41.

Macedo MO, Resende AS, Garcia PC, Boddey RM, Jantalia CP, Urquiaga S, Campello EFC, Franco AA (2008) Changes in soil C and N stocks and nutrient dynamics 13 years after recovery of degraded land using leguminous nitrogen-ﬁxing trees. Forest Ecology and Management 255: 1516–1524.

Marín-Spiotta E, Swanston CW, Torn MS, Silver WL, Burton SD (2008) Chemical and mineral control of soil carbon turnover in abandoned tropical pastures. Geoderma 143: 49–62.

Ngo KM, Turner BL, Muller-Landau HC, Davies SJ, Larjavaara M, Hassan NFN, Lum S (2013) Carbon stocks in primary and secondary tropical forests in Singapore. Forest Ecology and Management 296: 81–89.

Nurulita Y, Adetutu EM, Gunawan H, Zul D, Ball AS (2016) Restoration of tropical peat soils: The application of soil microbiology for monitoring the success of the restoration process. Agriculture, Ecosystems and Environment 216: 293–303.

Parrotta JA (1992) The role of plantation forests in rehabilitating degraded tropical ecosystems. Agriculture, Ecosystems and Environment 41: 115-133.

Parrotta JA, Knowles OH (2001) Restoring tropical forests on lands mined for bauxite: Examples from the Brazilian Amazon. Ecological engineering 17:219-239.

Powers JS, Haggara JP, Fisher RF (1997) The effect of overstory composition on understory woody regeneration and species richness in 7-year-old plantations in Costa Rica. Forestry Ecology and Management 99: 43-54.

Rhoades CC, Eckert GE, Coleman DC (1998) Effect of Pasture Trees on Soil Nitrogen and Organic Matter: Implications for Tropical Montane Forest Restoration. Restoration Ecology 6: 262–270.

Ribeiro MBN, Bruna EM, Mantovani W (2010) Inﬂuence of Post-Clearing Treatment on the Recovery of Herbaceous Plant Communities in Amazonian Secondary Forests. Restoration Ecology 18: 50–58.

Richards AE, Dalal RC, Schmidt S (2007) Soil carbon turnover and sequestration in native subtropical tree plantations. Soil Biology & Biochemistry 39: 2078–2090.

Ruiz-Jaen MC, Aide TM (2006) An integrated approach for measuring urban forest restoration success. Urban Forestry & Urban Greening 4: 55–68.

**Sang PM, Lamb D, Bonner M, Schmidt S (2013) Carbon sequestration and soil fertility of tropical tree plantations and secondary forest established on degraded land. Plant Soil 362:187–200.**

**Sierra CA, Valle JI, Orrego SA, Moreno FH, Harmon ME, Zapata M, Colorado GJ, Herrera MA, Lara W, Restrepo DE, Berrouet LM, Loaiza LM, Benjumea JF (2007) Total carbon stocks in a tropical forest landscape of the Porce region, Colombia. Forest Ecology and Management 243: 299–309.**

**Singh MK,** Astley H, Smith P, Ghoshal N **(2015)** Soil CO2-C flux and carbon storage in the dry tropics: Impact of land use change involving bioenergy crop plantation. **Biomass and Bioenergy 83: 123–130.**

**Singh K, Singh B, Singh RR (2012) Changes in physico-chemical,microbial and enzymatic activities during restoration of degraded sodic land: Ecological suitability of mixed forest over monoculture plantation. Catena 96: 57–67.**

**Singh KP, Mandal TN, Tripathi SK (2001) Patterns of restoration of soil physicochemical properties and microbial biomass in different landslide sites in the sal forest ecosystem of Nepal Himalaya. Ecological Engineering 17: 385–401.**

Song BL, Yan MJ, Hou H, Guan JH, Shi WY, Li GQ, Du S (2016) Distribution of soil carbon and nitrogen in two typical forests in the semiarid region of the Loess Plateau, China. Catena 143: 159–166.

**Tondoh JE, Monin LM, Tiho S, Csuzdi C (2007) Can earthworms be used as bio-indicators of land-use perturbations in semi-deciduous forest? Biology and Fertility of Soils 43:585–592.**

**Tripathi N, Singh RS (2008) Ecological restoration of mined-out areas of dry tropical environment, India. Environmental Monitoring and Assessment 146:325–337.**

**Urquiza-Haas T, Dolman PM, Peres CA (2007) Regional scale variation in forest structure and biomass in the Yucatan Peninsula, Mexico: Effects of forest disturbance. Forest Ecology and Management 247: 80–90.**

**Valencia V, García-Barrios L, West P, Sterling EJ, Naeem S (2014) The role of coffee agroforestry in the conservation of tree diversity and community composition of native forests in a Biosphere Reserve. Agriculture, Ecosystems and Environment 189: 154–163.**

Viana Junior AB, Souza VB, Reis YT, Marques-Costa AP (2014) Termite assemblages in dry tropical forests of Northeastern Brazil: Are termites bioindicators of environmental disturbances? Sociobiology 61(3): 324-331.

**Vieira DLM, Scariot A, Sampaio AB, Holl KD (2006) Tropical dry-forest regeneration from root suckers in Central Brazil. Journal of Tropical Ecology 22:353–357.**

**Volpato GH, Prado VM, Anjos L (2010) What can tree plantations do for forest birds in fragmented forest landscapes? A case study in southern Brazil. Forest Ecology and Management 260: 1156–1163.**

Wang F, Zhu W, Xia H, Fu S, Li Z (2010) Nitrogen Mineralization and Leaching in the Early Stages of a Subtropical Reforestation in Southern China. Restoration Ecology 18: 313–322.

**Wangpakapattanawong P, Kavinchan N, Vaidhayakarn C, Schmidt-Vogt D, Elliott S (2010) Fallow to forest: Applying indigenous and scientiﬁc knowledge of swidden cultivation to tropical forest restoration. Forest Ecology and Management 260: 1399–1406.**

**Werner FA, Gradstein SR (2009) Diversity of dry forest epiphytes along a gradient of human disturbance in the tropical Andes. Journal of Vegetation Science 20: 59-68.**

**Xiao K,** Heb T, Chena H, Penga W, Songa T, Wanga K, Lia D **(2017)** Impacts of vegetation restoration strategies on soil organic carbonand nitrogen dynamics in a karst area, southwest China. **Ecological Engineering 101: 247–254.**

**Zahawi RA, Augspurger CK (1999) Early Plant Succession in Abandoned Pastures in Ecuador. Biotropica 31: 540-552.**

**Zahawi RA, Holl KD, Cole RJ, Reid JL (2013) Testing applied nucleation as a strategy to facilitate tropical forest recovery. Journal of Applied Ecology 50: 88–96.**

**Zhuang X (1997) Rehabilitation and development of forest on degraded hills of Hong Kong. Forestry Ecology and Management 99: 197–201.**
