# Supplementary material for: Restoration of ecosystem services in tropical forests: A global meta-analysis
Source: PLoS One. 2018 Dec 27;13(12):e0208523. doi: 10.1371/journal.pone.0208523 (PMC6307725; doi:10.1371/journal.pone.0208523)
Supplement: S1 Fig — (DOCX) [file pone.0208523.s003.docx]

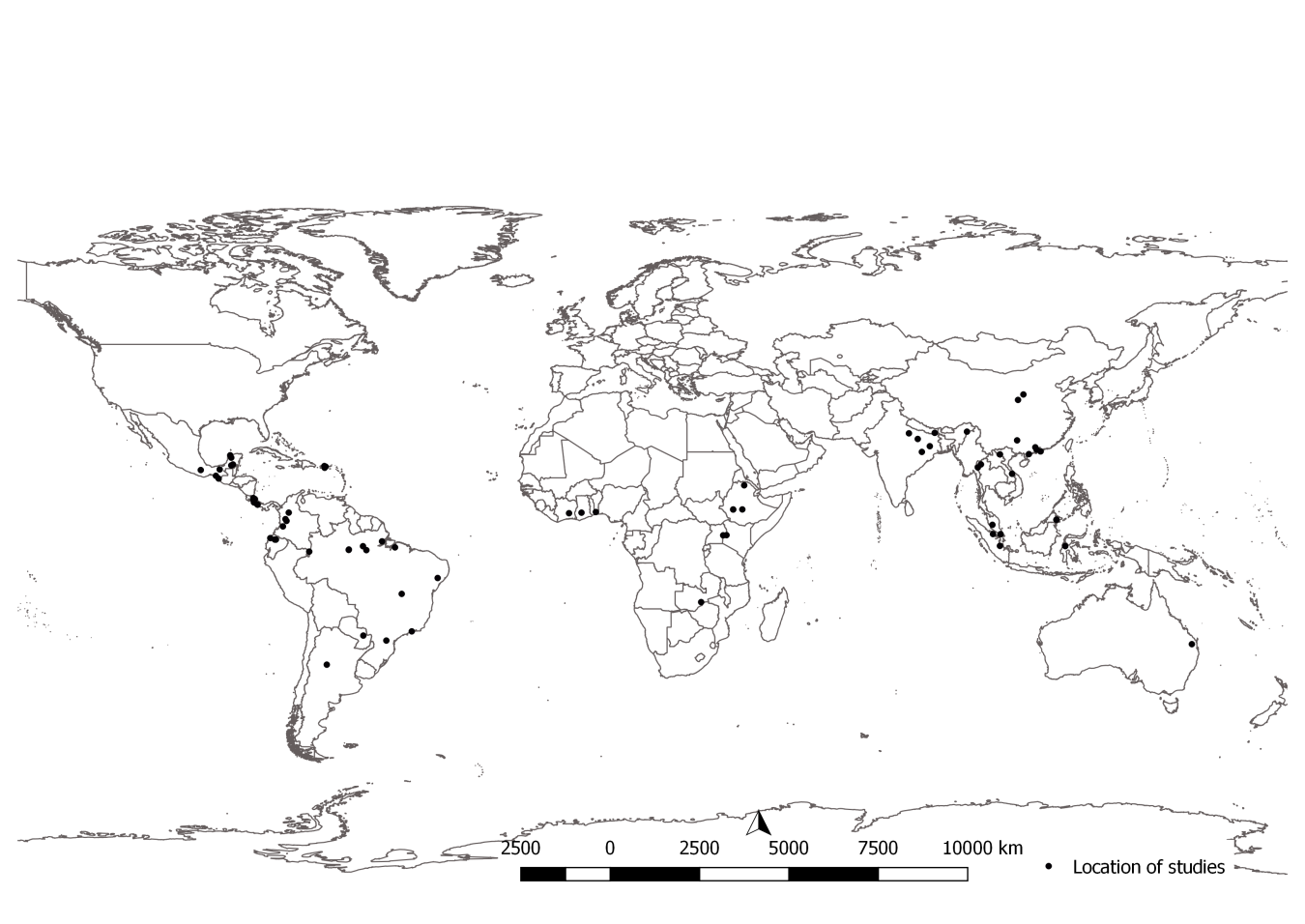


S1 Figure. Location of 69 studies from 25 countries distributed in five continents. Map from: Natural Earth (public domain): http://www.naturalearthdata.com/
