# Supplementary figures and images for: Restoration of ecosystem services in tropical forests: A global meta-analysis
Source: PLoS One. 2018 Dec 27;13(12):e0208523. doi: 10.1371/journal.pone.0208523 (PMC6307725; doi:10.1371/journal.pone.0208523)

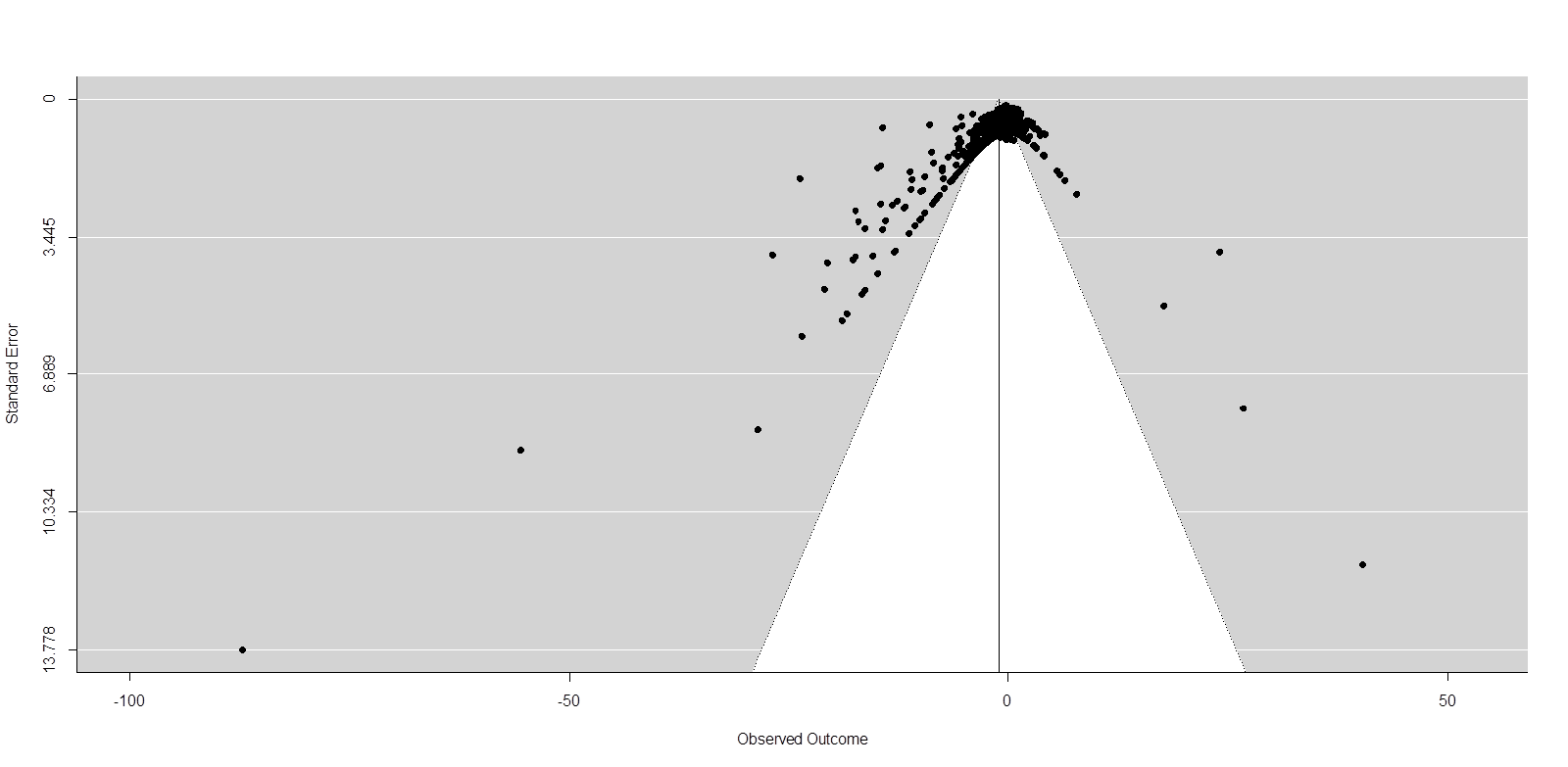


**S2 Figure. Funnel plot for comparison between restored and reference ecosystem.**

Supplement: S2 Fig — (DOCX) [file pone.0208523.s004.docx]

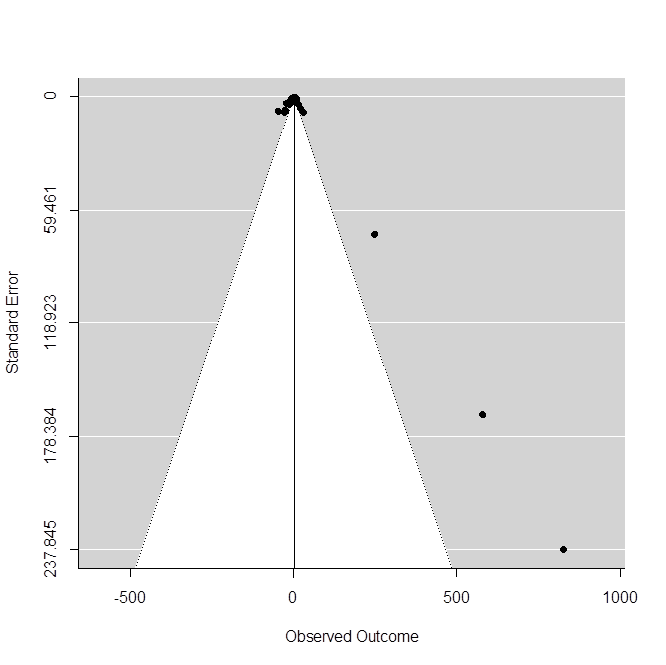


S3 Figure. Funnel plot for comparison between restored and degraded ecosystem.

Supplement: S3 Fig — (DOCX) [file pone.0208523.s005.docx]
